# Gefitinib Plus Interleukin-2 in Advanced Non-Small Cell Lung Cancer Patients Previously Treated with Chemotherapy

**DOI:** 10.3390/cancers6042035

**Published:** 2014-09-30

**Authors:** Melissa Bersanelli, Sebastiano Buti, Roberta Camisa, Matteo Brighenti, Silvia Lazzarelli, Giancarlo Mazza, Rodolfo Passalacqua

**Affiliations:** 1 Oncology Unit, University Hospital of Parma, Via Gramsci, 14, 43126 Parma, Italy; E-Mails: sebabuti@libero.it (S.B.); rcamisa@ao.pr.it (R.C.); 2 Oncology Unit, Azienda Istituti Ospitalieri di Cremona, Largo Priori, 1, 26100 Cremona, Italy; E-Mails: m.brighenti@ospedale.cremona.it (M.B.); s.lazzarelli@ospedale.cremona.it (S.L.); r.passalacqua@ospedale.cremona.it (R.P.); 3 Radiology Division, Spedali Civili di Brescia, P.le Spedali Civili,1, 25123 Brescia, Italy; E-Mail: radiologia1@spedalicivili.brescia.it

**Keywords:** gefitinib, interleukin-2, IL-2, lung cancer, non-small cell lung cancer, NSCLC, immunotherapy, tyrosine kinase inhibitors, TKI

## Abstract

The activation of lymphocytes by gefitinib treatment has been described. In this phase II pilot trial, we explored the possible synergism between IL-2 and gefitinib for non-small cell lung cancer (NSCLC) treatment. From September, 2003, to November, 2006, 70 consecutive patients with advanced, progressive NSCLC, previously treated with chemotherapy, received oral gefitinib 250 mg daily. The first 39 patients received gefitinib alone (G group). The other 31 also received subcutaneous IL-2 (GIL-2 group): 1 MIU/m^2^ (Million International Unit/m^2^)twice a day on Days 1 and 2, once a day on Days 3, 4, 5 every week for four consecutive weeks with a four-week rest period. Median follow-up was 25.2 months. Grade 3–4 toxicity of gefitinib was represented by skin rash (7%), asthenia/anorexia (6%) and diarrhea (7%); patients treated with IL-2 showed grade 2–3 fever (46%), fatigue (21%) and arthralgia (13%). In the GIL-2 group and G-group, we respectively observed: an overall response rate of 16.1% (6.4% complete response) and 5.1% (only partial response); a disease control rate of 41.9% and 41%; a median time to progression of 3.5 (CI 95% = 3.2–3.8) and 4.1 (CI 95% = 2.6–5.7) months; a median overall survival of 20.1 (CI 95% = 5.1–35.1) and 6.9 (CI 95% = 4.9–8.9) months (*p* = 0.002); and an actuarial one-year survival rate of 54% and 30%. Skin toxicity (*p* < 0.001; HR = 0.29; CI 95% = 0.16–0.54) and use of IL-2 (*p* < 0.001; HR = 0.33; CI 95% = 0.18–0.60) were independently associated with improvement of survival. In this consecutive, non-randomized, series of advanced NSCLC patients, the use of IL-2 increased the efficacy of gefitinib.

## 1. Introduction

Gefitinib (Iressa) is an oral selective epidermal growth factor receptor (EGFR) tyrosine kinase inhibitor (TKI) nowadays approved for the first line treatment of non-small-cell lung cancer (NSCLC) in the presence of EGFR gene mutations and also for NSCLC patients without activating mutations in further lines of treatment, on the basis of three pivotal phase III trials [1,2,3,4], more recently supported by further randomized controlled trials on naive patients harboring EGFR mutations [5,6]. Gefitinib was the first oral EGFR-TKI to become commercially available; at the beginning of the present study, it was clinically available only for compassionate use.

Interleukin-2 (IL-2), a cytokine signaling molecule necessary for growth, proliferation and differentiation of T-lymphocytes, has demonstrated antitumor activity in renal cell cancer and melanoma; previous studies on patients with NSCLC treated with IL-2 reported relatively long survival [7,8,9,10].

The imbalance of the IL-2/IL-2 receptor system in advanced NSCLC, with the decline in IL-2 levels and the significantly high soluble IL-2 receptor (sIL-2R) concentrations, has been evidenced and suggested to represent a marker of disease with potential prognostic value [11]; on the other hand, the role of IL-2 activation in the restoration of the immunocompetence of lymphocytes against lung cancer has been demonstrated [12].

The strategy of harnessing the immune system, previously relatively abandoned in lung cancer treatment, has actually had a rebound of interest with the emerging of new therapeutic targets in immunotherapy [13,14,15,16]. Vaccination strategies are also promising: a phase IIB/III study is currently ongoing to evaluate the efficacy of TG4010 (recombinant vaccinia virus of the Ankara strain), a genetically modified virus expressing both mucinous glycoprotein 1 (MUC1) and IL-2, in advanced NSCLC patients [17]. In this renewed focus on the use of immunotherapy in lung cancer, in particular when combined with standard therapies in order to potentiate their efficacy, also T-lymphocytes and their activating cytokines have been reconsidered [18]. Combination therapy of EGFR-TKI with cytokines has recently been experimented upon with some positive results in order to reverse resistance to TKI in NSCLC [19,20]. Recent evidence indicated that lymphocytes are activated by gefitinib treatment via activation of platelets, which release serum regulated on activation normal T-cell expressed and secreted (RANTES) and regulate chemokine secretion, inducing lymphocyte migration. Moreover, plasma soluble P-selectin, RANTES and serum sIL-2R levels increased significantly in patients receiving gefitinib [21,22]. Other authors found that EGFR-TKI affects the cancer related networks of pro-inflammatory cytokines and activates the lymphocytic responses; this evidence suggests a possible synergism between the EGFR molecular pathway inhibition and immune system modulation in promoting tumor shrinkage [23,24].

In this pilot study, we explored the possible synergism between IL-2 and gefitinib in terms of response rate, survival and toxicity, with the purpose of evaluating the efficacy of this association in advanced NSCLC.

## 2. Patients and Methods

### 2.1. Patients Selection

Eligibility criteria included: age ≥18 years; histologic or pathologic confirmation of NSCLC; prior treatment with at least one chemotherapy regimen for recurrent or metastatic disease, including at least one platinum-based combination; measurable disease by the Response Evaluation Criteria In Solid Tumors (RECIST) Group response criteria [25]; and an Eastern Cooperative Oncology Group Performance Status (ECOG PS) from 0 to 2. Patients were required to have adequate baseline hematologic (absolute neutrophil count ≥1,500/μL, platelets ≥100,000/μL and hemoglobin ≥8 g/dL), renal (serum creatinine level ≤2.0 mg/dL or calculated creatinine clearance ≥50 mL/min) and hepatic (alkaline phosphatase, AST and ALT levels ≤2.5 × upper limit of normal and total bilirubin ≤1.5 × upper limit of normal) function parameters. Patients with asymptomatic brain metastases who were adequately treated at least 3 weeks before enrollment and were not receiving corticosteroids were eligible. Cases of prior treatment with any other experimental drug within two months since this study entry were excluded. Patients with serious autoimmune disease or a significant history of cardiac disease and patients undergoing major thoracic or abdominal surgery within 30 days before the first administration of therapy were also excluded. Prior radiation therapy was to be completed at least 3 weeks before enrollment and could not have been given to the only site of measurable disease unless there was documentation of disease progression post-irradiation. Prior chemotherapy must have been completed at least 30 days before the first administration of experimental treatment. A 12-lead electrocardiogram (ECG), a chest radiography (RX) and a chest and abdomen computed tomography (CT) scan were performed within 28 days before treatment. A bone scintigraphy, a CT scan or magnetic resonance (MR) imaging of the brain was also performed within 28 days before experimental treatment at the physician’s discretion; other examinations were performed if considered necessary to the evaluation of lesions. Medical history, physical examination, assessment of ECOG PS, cell blood count differential, platelet count and a chemistry panel were completed within 14 days of treatment. A negative urine or serum pregnancy test was required for women of childbearing potential within 72 h before treatment. The study had the approval of the local ethical committee; written informed consent was provided for each patient.

### 2.2. Study Design and Treatment Plan

From September, 2003, to November, 2006, 70 consecutive eligible chemotherapy pretreated patients with advanced, progressive NSCLC were included in this study and planned to receive oral gefitinib 250 mg daily in a compassionate use program at a single institution, in the Oncology Unit of Istituti Ospitalieri of Cremona (Italy). This is a phase II, non-randomized, pilot trial combined with a cohort design, each having a different outcome event. The phase II portion was a Fleming one-stage design used to determine the sample size for estimating the response rate as the primary endpoint; it was decided to set *p_0_* = 0.5 (expected response rate with only gefitinib therapy in non-selected pretreated advanced NSCLC) and *p_A_* = 0.10 (expected response rate with gefitinib plus IL-2). Considering a one-sided significance level test *α* = 0.05 with 80% power (β = 0.2), the number of patients to accrue were 31: if the number of treatment response were ≥5, the null hypothesis H_0_ was rejected: *p_A_* ≥ *p_0_*. For the first 39 consecutive patients (cohort portion), the administration of gefitinib alone (G group) was scheduled until disease progression or intolerable toxicity, and no specific design was applied to this group, even if the data were prospectively collected; the remaining 31 consecutive patients were intended to constitute a separate group (phase II portion) receiving gefitinib in association with subcutaneous IL-2 (GIL-2 group), according to the following eight-week schedule (Table 1): 1 MIU/m^2^ twice a day on Days 1 and 2, once a day on Days 3, 4, 5 every week for 4 consecutive weeks with a 4-week rest period until disease progression or intolerable toxicity. A premedication with paracetamol (500 to 1,000 mg) was permitted before the administration of IL-2. Aside from disease progression, treatment could be suspended at any time on the investigators’ opinion for the appearance of serious adverse events or unexpected reactions or for patient’s refusal; these cases were equally considered as assessable. Temporary discontinuation and/or dose reduction of 50% of one or both drugs was allowed for whatever toxicity at least of Grade 3.

**Table 1:** Schedule of IL-2 treatment repeated for 4 consecutive weeks with a 4-week rest period until progression of the disease or intolerable toxicity.

Day of Week	1	2	3	4	5	6	7
**IL-2 bid**	x	x					
**IL-2/die**			x	x	x		

IL-2 bid: subcutaneous Interleukin-2 one million IU/m^2^ twice a day. IL-2/die: subcutaneous Interleukin-2 one million IU/m^2^ once a day.

### 2.3. Assessments

The above described pretreatment evaluations were planned. Physical and laboratory examinations were performed every 28 days. Response assessments based on RECIST criteria [25] were scheduled every 10–12 weeks (or before, in the case of clinical suspicion of disease progression). The cases of clear clinical progression were considered as disease progression, even in the absence of radiological assessment when not feasible. Responses had to be confirmed by an external radiologist with repeated assessments no less than 4–6 weeks after the initial claim of disease response. Time to progression (TTP) was defined as the time from the beginning of treatment to disease progression (or death, if it was first). Overall survival (OS) was defined as the time from the beginning of the treatment to death for any cause. All adverse reactions were assessed according to National Cancer Institute Common Toxicity Criteria (version 3.0) [26]. In the case of Grade 3 acne-like rash (rash/desquamation) or diarrhea, gefitinib therapy was discontinued for up to four consecutive weeks. If the toxicity resolved to a grade ≤2 by the following period, treatment could have been resumed.

### 2.4. Statistical Analysis

The primary endpoint of this study was to the assess response rate (RR), intended as the sum of partial and complete responses according to RECIST criteria [25], of combination therapy with gefitinib and IL-2; the secondary endpoints were the evaluation of survival, TTP and safety with the exploratory comparison between the two groups of treatment. The analysis of data has been conducted according to the principle of the “intention-to-treat”. The product of the limit method (Kaplan-Meier) has been used for the curve of survival and the log-rank test for the comparison among the curves. Models of regression (Cox) have been adopted for the analysis of the prognostic factors.

## 3. Results

### 3.1. Patients Characteristics

From September, 2003, to November, 2006, 70 consecutive patients with advanced, previously treated progressive NSCLC received treatment according to this study plan: 39 patients were in the G group, while 31 were in the GIL-2 group. All patients were EGFR-TKI naive. Patient demographics and disease characteristics are summarized in Table 2.

**Table 2:** Patients’ demographics and disease characteristics.

PARAMETER	Gefitinib Only No. of Patients	Gefitinib + IL-2 No. of Patients	AllPatients
Demographic characteristics			
Number of patients	**39 (56%)**	**31 (44%)**	**70**
Males	28 (72%)	17 (55%)	45 (64%)
Females	11 (28%)	14 (45%)	25 (36%)
Age, years			
Median	71	69	70
Range	39–84	44–80	39–84
ECOG PS			
0	14 (36%)	17 (55%)	31 (44%)
1	17 (44%)	13 (42%)	30 (43%)
≥ 2	8 (20%)	1 (3%)	9 (13%)
Smoking History			
Yes	35 (90%)	23 (74%)	58 (83%)
No	3 (8%)	7 (23%)	10 (14%)
Unknown	1 (2%)	1 (3%)	2 (3%)
Histology			
Adenocarcinoma	24 (62%)	24 (77%)	48 (69%)
Squamous cell carcinoma	5 (13%)	3 (10%)	8 (11%)
Bronchoalveolar-cell	2 (5%)	2 (6%)	4 (6%)
Undifferentiated	8 (20%)	2 (6%)	10 (14%)
Number of disease sites			
1–2	12 (31%)	9 (29%)	21 (30%)	
≥3 (range 3–8)	27 (69%)	22 (71%)	49 (70%)	
Lung disease				
Yes	37 (95%)	30 (97%)	67 (96%)	
No	2 (5%)	1 (3%)	3 (4%)	
Liver metastasis				
Yes	10 (26%)	1 (3%)	11 (16%)	
No	29 (74%)	30 (97%)	59 (84%)	
Bone metastasis				
Yes	12 (31%)	12 (39%)	24 (34%)	
No	27 (69%)	19 (61%)	46 (66%)	
Node metastasis				
Yes	26 (66%)	18 (58%)	44 (63%)	
No	13 (33%)	13 (42%)	26 (37%)	
Adrenal metastasis				
Yes	5 (13%)	4 (13%)	9 (13%)	
No	34 (87%)	27 (87%)	61 (87%)	
Brain metastasis				
Yes	8 (21%)	5 (16%)	14 (20%)	
No	31(79%)	26 (84%)	56 (80%)	
Previous Chemotherapy for metastatic disease				
One line	31 (79%)	22 (71%)	53 (76%)	
Two lines	6 (15%)	7 (23%)	13 (18%)	
Three lines	2 (5%)	2 (6%)	4 (6%)	
Previous platinum therapy				
Yes	34 (87%)	30 (97%)	64 (91%)	
No	5 (13%)	1 (3%)	6 (9%)	
Further therapy after progressive disease on treatment study				
Yes	9 (23%)	13 (42%)	22 (31%)	
No	30 (77%)	18 (58%)	48 (69%)	

ECOG PS: Eastern Cooperative Oncology Group Performance Status.

Two thirds of patients were male. The median age was 70 years (range 39 to 84 years). Thirty one patients (44%) had a baseline ECOG PS = 0, 30 (43%) PS = 1 and nine (13%) PS ≥ 2. Fifty-three (76%) patients received only one prior chemotherapy regimen, while 17 (24%) received ≥2 prior lines of treatment. Forty-eight (69%) patients had adenocarcinoma, eight (11%) had squamous cell carcinoma, and four (6%) had bronchoalveolar-cell carcinoma subtype; the remaining 10 (14%) had undifferentiated NSCLC. Only 10 (14%) patients presented a never-smoking history. According to the intention-to-treat principle, all patients have been considered in the evaluation of TTP, OS and toxicity. The median duration of the follow-up was 25.2 months (range: from 8.3 to 39.4 months): 27.5 months (range 8.3–36.9) and 21.8 months (range 8.8–39.4) for the G and GIL-2 groups, respectively.

### 3.2. Responses, Effectiveness and Survival

At the end of the observation period (November, 2006), 17 (24%) patients were still alive. All patients were considered evaluable for response; two complete responses (CR, 3%) and five partial responses (PR, 7%) were recorded in the overall population, with an objective RR of 10%; 22 patients had stable disease (SD, 31.4%) as the best response. The overall disease control rate was 41.4%, and the actuarial one-year survival rate was 40%. Median TTP was 3.7 months (CI 95% 2.7–4.3) and median overall survival (OS) was 7.9 months (CI 95% 4.9–10.8).

**Figure 1:**
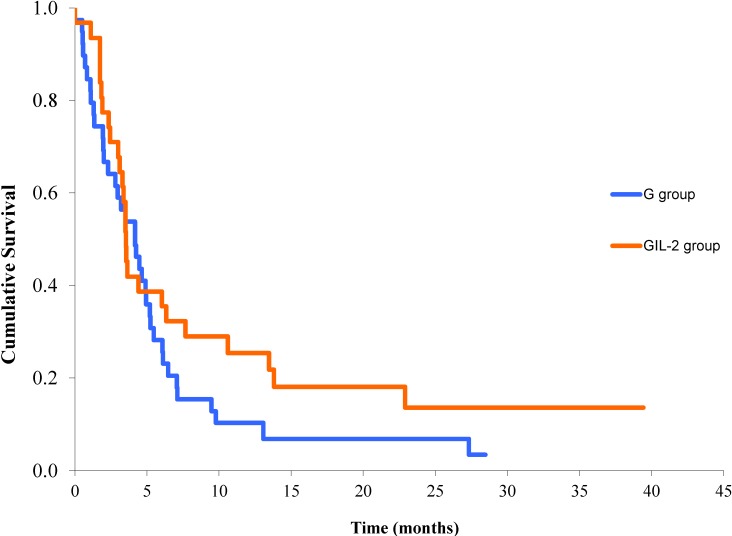
Time to progression in G (gefitinib) and GIL-2 (gefitinib and IL-2) groups (*p* = not significant).

When comparing patients who received IL-2 and gefitinib with patients who received gefitinib alone, we respectively observed an objective RR of 16.1% (with two cases of CR, 6.4% of GIL-2 patients) and 5.1% (with only partial response in the G group). The disease control rate was 41.9% and 41%, respectively. The median TTP was 3.5 (CI 95% 3.2–3.8) and 4.1 months (CI 95% 2.6–5.7) in the GIL-2 and G groups, respectively, without reaching statistical significance (Figure 1).

**Figure 2:**
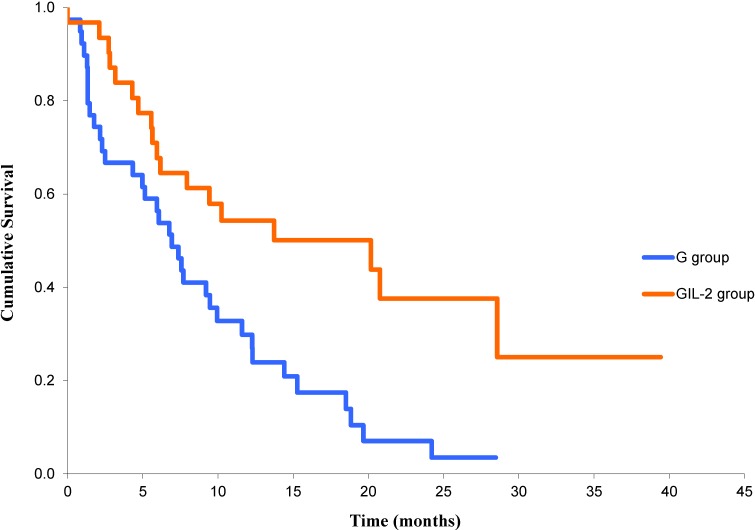
Overall survival in G and GIL-2 groups (*p* = 0.002).

The median OS in the GIL-2 group was 20.1 months (CI 95% 5.1–35.1), while in the G group, it was 6.9 months (CI 95% 4.9–8.9) (Figure 2), with statistical significance in favor of the combined treatment (*p* = 0.002) and actuarial one-year survival rates of 54% and 30%, respectively.

Multivariate analysis according Cox’s regression, including PS, gender, smoking, skin toxicity of any grade and use of IL-2, revealed that only skin toxicity (*p* < 0.001; HR 0.29; CI 95% 0.16–0.54) and use of IL-2 (*p* < 0.001; HR 0.33; CI 95% 0.18–0.60) were significantly associated with improvement in OS independently of other factors.

### 3.3. Toxicity

Toxicity was assessed according to National Cancer Institute Common Toxicity Criteria (version 3.0) [25] (see Table 3).

**Table 3:** Overall population toxicity.

Toxicity	Events(No. of Patients, %)	WHO grade (No. of Patients, %)			
		Grade 1	Grade 2	Grade 3	Grade 4
Rash	31 (44.3%)	8 (11.4%)	18 (25.7%)	4 (5.7%)	1 (1.4%)
Asthenia	18 (25.7%)	8 (11.4%)	7 (10%)	3 (4.3%)	0
Anorexia	15 (21.4%)	9 (12.9%)	5 (7.1%)	1 (1.4%)	0
Diarrhea	12 (17.1%)	7 (10%)	0	5 (7.1%)	0
Dyspnea	8 (11.4%)	6 (8.6%)	2 (2.9%)	0	0
Transaminase alteration	4 (5.7%)	3 (4.3%)	0	1 (1.4%)	0
Fever	18 (25.7%)	4 (5.7%)	14 (20%)		0
Fatigue	7 (10%)	0	7 (10%)		0
Arthralgias	4 (5.7%)	0	4 (5.7%)		0

Gefitinib was generally well-tolerated: five (7%) patients had Grade 3–4 skin toxicity, four (6%) had Grade 3 asthenia/anorexia and five had Grade 3 diarrhea. In four cases, gefitinib was permanently discontinued because of Grade 3–4 toxicity. Only two patients had gefitinib suspension due to causes other than toxicity or imaging documentation of the progression of disease: in one case, the suspension was related to the occurrence of new diseases (acute myocardial infarction and new primary cancer); in the other case, interruption was requested by the patient.

About IL-2 toxicity, most patients experienced erythema and induration at the IL-2 injection site and constitutional symptoms, such as fever, chills, fatigue and malaise; these symptoms were present only during the days of IL-2 administration and completely regressed during the days of suspension and at the end of the IL-2 cycles. Main Grade 2–3 toxicity in the GIL-2 group was represented by fever in 46%, fatigue in 23% and arthralgias in 13% of patients, besides the gefitinib-related toxicity. Incidence and grade of IL-2 toxicity were independent from the association with gefitinib or not (in cases of temporary discontinuation of gefitinib); moreover, in the same patient, they tended to decrease with the succession of the cycles. Permanent discontinuation of treatment due to toxicity occurred in three cases in the G group and in only one patient of the GIL-2 group.

## 4. Discussion and Conclusions

In the current phase II pilot trial, a new molecular therapy with consolidated activity in advanced NSCLC, such as gefitinib, has been associated with an immunotherapy treatment, IL-2, currently considered obsolete when used as single agent in this setting. IL-2 has in fact been studied in the past with poor results in patients with solid tumors other than melanoma and renal cell carcinoma, lacking randomized trials in NSCLC and making difficult the possibility of drawing reliable conclusions on the issue [27]. Its activity in lung cancer has nevertheless been suggested by encouraging evidence in neoadjuvant and adjuvant settings [28], despite conflicting results from various studies, which hinder a definitive pronouncement on the use of IL-2 in advanced disease [7,8,9,10,29,30,31,32,33,34,35,36]. The possibility to reintroduce IL-2 in NSCLC treatment in order to potentiate the effectiveness of a TKI constitutes a novel strategy that can be supported by the recent interesting findings in the field of immunomodulation, both related to lung cancer and operated by gefitinib [11,22,23,24], as previously described in our Introduction, providing a rationale for this association.

The outcomes of the present trial in terms of RR are noteworthy: with the limit of the restricted number of patients, the RR in the GIL-2 group is threefold compared to that of gefitinib alone (16% *versus* 5%); moreover, only with IL-2 there were also CR among disease responses. Despite this amazing result, the disease control rate was similar in the two groups of treatment; also, in terms of TTP, no significant differences have been reached, suggesting that the more efficient tumor shrinkage obtained does not result in an increase of the disease control duration.

The independent predictive value for survival of gefitinib-related skin toxicity endorses also in this study the evidence that has already emerged from the recent literature, which confirmed skin rash as a predictive and prognostic factor in NSCLC patients treated with anti-EGFR TKI [37,38].

The safety profile of gefitinib was shown not to be affected by the association with IL-2; in fact, the overall rates of toxicity of any grade in terms of skin rash, diarrhea, asthenia and anorexia do not exceed those reported in the pivotal trials [1,2], even better evidencing a good tolerability with a low incidence of Grade 3–4 adverse events. On the other hand, the toxicity profile of IL-2 with the schedule used in our study demonstrated this to be easily manageable and often overcome in the course of successive cycles of therapy, allowing the combination therapy without significantly affecting the overall tolerability of treatment.

The secondary endpoint, represented by OS, with the limit of a non-randomized trial, is greatly achieved, reaching statistical significance and obtaining an almost tripled survival with the association of IL-2 to gefitinib with respect to the controls with gefitinib alone (20 *versus* 7 months), whose OS is aligned with those reported for gefitinib in phase III randomized trials in pretreated, unselected patients [2,3,4]. Nevertheless, due to imbalances in patients’ characteristics between the two groups and the small sample size of the study, the interpretation of survival curves is limited. Our undoubted limit of a non-selected population based on EGFR status is due to the fact that the role of this molecular predictive marker was not already demonstrated at the opening of this study. The potential benefit of administering IL-2 plus gefitinib in an unselected population loses real significance when most ongoing studies in lung cancer are currently investigating targeted agents in molecularly-selected patient populations. Our outcome results may be surely improved in populations harboring EGFR mutations, whilst the mutational status of our patients, treated with gefitinib at the time of the expanded access program, was unknown, but more robust data from larger, randomized studies are needed. Over and above the fault of an outdated study, other great limits of our trial are represented by the small sample size and the lack of randomization, with potential selection bias and imbalance of clinical characteristics and prognostic factors, which could have affected the outcome in the two groups: on the one hand, the incidence of EGFR-mutated tumors may have been favored in the GIL-2 group by a higher rate of female and non-smoker patients; on the other hand, a great number of patients with poor PS was reported for the G group. In addition, higher rates of further therapies after the study treatment in the GIL-2 group might also have influenced the survival results.

In conclusion, the results of this phase II pilot study suggest that the addition of IL-2 may improve the objective RR of gefitinib monotherapy in advanced NSCLC, with some tolerable adverse events caused by IL-2, thus maintaining a good safety profile of treatment. Further investigations with randomized controlled trials would be needed to definitely verify the efficacy of these drugs associations for NSCLC patients.
